# Research on Effectiveness of Prior Cancer on Survival Outcomes for Patients with Nonmetastatic Triple-Negative Breast Cancer: A Competing Risk Analysis and Propensity Score Matching Analysis of the SEER Database

**DOI:** 10.1155/2021/9988624

**Published:** 2021-09-17

**Authors:** Heyan Chen, Lutong Yan, Shengyu Pu, Lizhe Zhu, Huimin Zhang, Can Zhou

**Affiliations:** Department of Breast Surgery, The First Affiliated Hospital of Xi'an Jiaotong University, Xi'an, China

## Abstract

**Introduction:**

Knowledge of the effect of prior cancer on long-term survival outcomes for patients with nonmetastatic triple-negative breast cancer (TNBC) remained unclear. The aim of this study was to explore and identify the effectiveness of prior cancer on breast cancer-specific death (BCSD) and other cause-specific death (OCSD) in patients with nonmetastatic TNBC.

**Materials and Methods:**

Data of 29,594 participants with nonmetastatic TNBC patients were extracted from the Surveillance, Epidemiology, and End Results (SEER) database from 2010 to 2016. Prognostic predictors were identified by propensity score matching (PSM) analysis combined with univariate cumulative incidence function (CIF) and multivariate Fine and Gray competitive risk analyses.

**Results:**

Among the women with nonmetastatic TNBC included in the unmatched cohort, a total of 5,375 (18.2%) subjects had prior cancers (P-TNBC) and 24,219 (81.8%) had no prior cancer (NP-TNBC). Patients with P-TNBC tended to have poorer BCSD (Gray's test, *p*=0.0131) and OCSD (Gray's test, *p*=0.0009) in comparison with those with NP-TNBC after PSM. However, the risk of BCSD (*p*=0.291) and OCSD (*p*=0.084) found no difference among P-TNBC patients with one prior cancer and two or more prior cancers after PSM. Additionally, subjects with younger age, advanced T stage, advanced N stage, and advanced differentiation grade tumors were likely to develop BCSD, whereas those with breast-conserving surgery (BCS), radiotherapy, or chemotherapy tended to have a lower incidence of BCSD.

**Conclusion:**

Our study demonstrated that prior cancer was related to the worse BCSD and OCSD rate and could be identified as a reliable survival predictor for patients with nonmetastatic TNBC. This study may provide some reference value for the treatment mode of TNBC patients with prior cancer in the future.

## 1. Introduction

A quarter of all global deaths have been attributed to cancers; however, the cancer-related death rate declined significantly from 1991 to 2018 (by 22%) [1]. The growth in the number of cancer survivors is due to the development of diagnostic technologies (including screening) and the application of new drugs [2]. However, the improvement in survival undoubtedly contributed to increased cumulative incidences of multiple primary cancers (MPCs) [3, 4] and corresponding histories of prior cancer, the prognosis of which was influenced differently by diverse tumor types [5]. Previous studies reported that about 4%–14% of patients with triple-negative breast cancer (TNBC) had a previous history of cancer [6, 7]. Accordingly, TNBC was regarded as one prognostic factor in patients with first primary cancer [8].

Triple-negative breast cancer (TNBC), which accounted for 15%–20% of all breast cancer patients [9], was a kind of breast cancer that lacked expression of estrogen receptor, progesterone receptor, or human epidermal growth factor receptor type 2 (HER2) [10]. TNBC seemed like a highly invasive and heterogeneous tumor, usually manifested as high-grade invasive ductal carcinoma and often accompanied by distant metastasis, with a higher rate of early recurrence and poor prognosis compared with other breast cancer subtypes [11]. However, due to the lack of clinical trials on TNBC as a second or third primary cancer patient or even more primary cancer patients, the prognosis of TNBC as a multiple primary cancer patient had not been evaluated until now. The real-world effectiveness of prior cancer on the long-term prognosis in patients with nonmetastatic multiple primary TNBC deserved further study and discussion [12].

The purpose of this study was threefold. First, we determined whether prior cancer was an independent prognostic factor for BCSD in patients with TNBC. Second, we further explored whether there were differences in BCSD among TNBC patients with a history of one cancer or two or more cancers. Finally, we explored whether prior cancer history would affect the treatment decisions of patients with TNBC and whether different treatment decisions would affect the prognosis of such patients. Therefore, we conducted this study using the SEER database to determine the impact of prior cancer on BCSD and OCSD in patients with TNBC through PSM analysis. Then, we identified the prognosis factors of BCSD and OCSD through competitive risk analysis in patients with P-TNBC. Furthermore, the risk differences of BCSD and OCSD under different treatment modes were further investigated via combining PSM analysis with competitive risk analysis.

## 2. Materials and Methods

### 2.1. Data Sources

The SEER database is the largest public cancer dataset in the world and is maintained by the National Cancer Institute, which provides complete information, including patient demographics, cancer diagnosis, tumor characteristics, first course of treatment, and follow-up for vital status. Our study cohort was extracted from SEER*∗*Stata version 8.3.6 (SEER ID:14518-Nov 2018), which included population-based data from 18 cancer registries in about 30% of the US population from 1975 to 2016. Since the SEER database was publicly accessible to users worldwide, informed patient consent was not required for this study. Therefore, it was considered to be exempt from the review of the Ethics Committee of the First Affiliated Hospital of Xi'an Jiaotong University.

### 2.2. Patients and Variables Selection

Patients meeting the following criteria were included: (1) female patients; (2) diagnosed as TNBC between 2010 and 2016 (since HER2 status was only included in SEER data after 2010, candidates in this study were included between 2010 and 2016); (3) with primary cancer; and (4) diagnosed as M0 stage. Then, patients meeting the following criteria were excluded: (1) age less than 20 years; (2) unknown demographic features including race and marital status; (3) unknown or indefinite clinical information including site of laterality (bilateral, only one side but side unspecified or paired site but no information concerning laterality), T stage (excluding any T, mets, NA, Tx adjusted or T0 or Tis) and *N* stage (excluding NA and Nx adjusted); (4) unknown or no surgery information; and (5) patients with the follow-up type of autopsy/death certificate only. The following data were collected for each patient in this study: age, race, marital status, laterality, tumor differentiation grade, tumor size, lymph node status, radiotherapy status, chemotherapy status, surgery methods, survival months, and causes of death from the SEER database.

**Figure 1:**
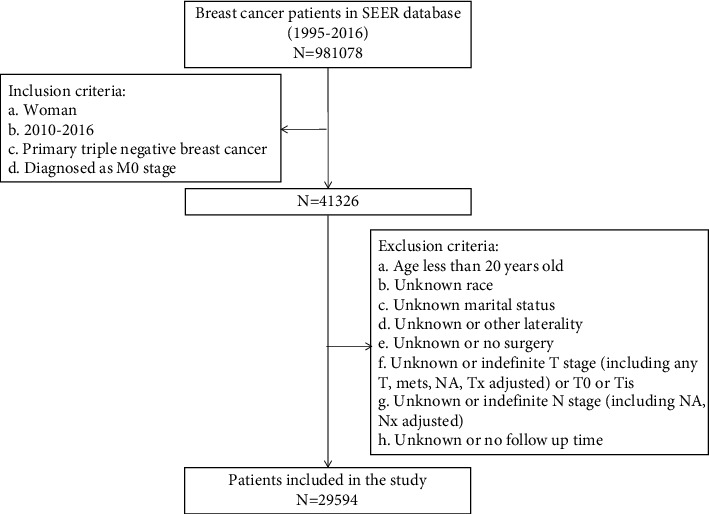
The flowchart of the included population in this study.

A total of 29594 patients were included, the flowchart of patient screening is shown in Figure 1. Prior cancer derived from the SEER sequence number, which described the sequence of all resectable malignancies during a patient's lifetime. The sequence number of “00” indicated that the patients had only one primary cancer in their lifetime. For subjects with MPCs, the sequence number of “01” suggested the first cancer, “02” suggested the second one, and so forth. In this study, for patients with TNBC, the sequence numbers of “00” and “01” were defined as no prior cancer (NP-TNBC) group, and the sequence numbers of “02,” “03,” “04,” and “05” were defined as prior cancer (P-TNBC) group.

### 2.3. Endpoints

The primary endpoint of this study was BCSD, which was referred to the time from the date of diagnosis to the date of death from breast cancer. The second endpoint of the study was OCSD, which was referred to the time from the date of diagnosis to the date of death from other causes.

### 2.4. Statistical Analysis

The Pearson chi-square test or Fisher's exact test was administrated to test the independence of patient demographics and treatment-related variables among groups. Categorical variables were reported as the number of cases and percentages. Propensity score matching (PSM) was used to match two groups of people on a one-to-one or one-to-many according to their propensity score. The PSM program and standardization difference were calculated by using the nearest-neighbor matching method with a caliper distance of 0.05 and R packages of “*MatchIt*” [13].

A competing risk model analysis was used to mitigate the estimation bias by classifying death causes into two subgroups. Firstly, the cumulative incidence function (CIF) was to evaluate the 1‐year, 3‐year, and 5‐year probabilities of BCSD and OCSD [14]. Secondly, in the multivariate survival competing risk analysis, we performed the Fine and Gray proportional distribution risk model to predict BCSD and OCSD by *R* package of “*cmprsk*” and “*riskRegression*” [15, 16]. Thirdly, Fine and Gray competitive risk regression was used to evaluate BCSD and OCSD in different treatment modes.

All statistical analyses were performed using *R* statistical software version 3.5.2. All statistical tests were two-sided, and the level of significance was set at *p* < 0.05.

## 3. Results

### 3.1. Characteristics between P-TNBC and NP-TNBC Patients

Patients were followed up until November 2018, and the median follow-up time was 39 months (ranging from 1 month to 83 months). In this study, a total of 29,594 female patients with TNBC were enrolled, of whom 5,375 (18.2%) had prior cancers of TNBC (P-TNBC) and 24,219 (81.8%) had no prior cancer of TNBC (NP-TNBC).

Before PSM analysis, the result showed that patients with P-TNBC were more common in elder adults more than 60 years old (*p* < 0.001), other race (*p* < 0.001), or single women (*p* < 0.001). Additionally, patients with P-TNBC had smaller tumor size (T1: 58.3% vs. 44.8%, *p* < 0.001), lower risk of lymph node infiltration (N0: 76.3% vs. 66.6%, *p* < 0.001), lower rate of breast-conserving surgery (BCS) (36.9% vs. 52.6%, *p* < 0.001), radiotherapy (32.9% vs. 67.1%, *p* < 0.001), and chemotherapy (60.6% vs. 76.8%, *p* < 0.001) than those with NP-TNBC (Table 1). There was no statistically significant difference in the distribution of baseline characteristics between the two groups after one-to-one matched PSM analysis (Table 1).

**Figure 2:**
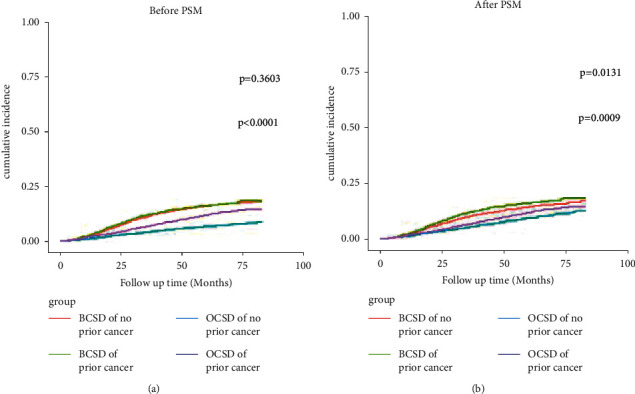
Cumulative incidence function analysis of TNBC with prior cancer and TNBC with no prior cancer before and after PSM analysis. BCSD: breast cancer-specific death; OCSD: other cause-specific death; PSM: propensity score matching. *p* value <0.05 was considered statistically significant.

Next, as can be seen from Figure 2, there was no difference in BCSD between P-TNBC and NP-TNBC patients (Gray's test, *p*=0.3603), but after PSM, there was a difference between P-TNBC and NP-TNBC patients (Gray's test, *p*=0.0131); that is, the BCSD rate for patients with P-TNBC was higher than that of those with NP-TNBC. This suggested that prior cancer was a poor prognostic factor in BCSD for patients with TNBC. In addition, whether before and after PSM, the OCSD rate for patients with P-TNBC was higher than those with NP-TNBC (Gray's test, *p* < 0.0001; *p*=0.0009), which suggested that prior cancer was also a poor prognostic factor for OCSD in patients with TNBC.

### 3.2. Characteristics between One Prior Cancer and Two or More Prior Cancers of P-TNBC Patients

We divided patients with P-TNBC into two groups (one prior cancer 85.3% vs. two or more prior cancers 14.7%) according to the number of prior cancers. The result indicated that, for patients with P-TNBC, one prior cancer was common in adult women of less than 69 years (*p* < 0.001), black or white women (*p*=0.001), and higher lymph node infiltration (N1, 17.0% vs. 10.45%; N2, 4.5% vs. 2.8%; N3, 3.6% vs. 2.8%, *p* < 0.001). Additionally, for patients with P-TNBC, patients with one prior cancer were more likely to receive BCS (37.8% vs. 31.2%, *p* < 0.001), radiotherapy (34.2% vs. 25.5%, *p* < 0.001), or chemotherapy (62.5% vs. 49.3%, *p* < 0.001) when compared to those with two or more prior cancers (Table 2). There was no statistically significant difference in the distribution of baseline characteristics between the two groups after one-to-one matched PSM analysis (Table 2).

**Figure 3:**
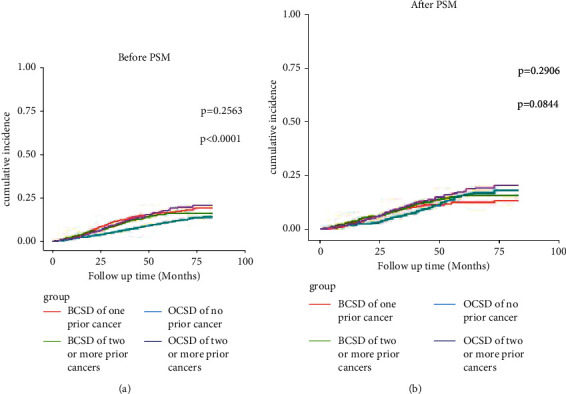
Cumulative incidence function analysis of one prior cancer and two or more prior cancers of P-TNBC patients before and after PSM analysis. P-TNBC: triple-negative breast cancer with prior cancers; BCSD: breast cancer-specific death; OCSD: other cause-specific death; PSM: propensity score matching. *p* value <0.05 was considered statistically significant.

Furthermore, as shown in Figure 3, we discovered there were no statistical differences in the risk of BCSD between P-TNBC patients with one prior cancer and two or more prior cancers (*p*=0.256) (Figure 3). While the risk of OCSD was found to have statistical differences between the two groups, the higher the amount of cancer history is, the more likely the OCSD was to occur (*p* < 0.001) (Figure 3). However, after PSM analysis, no difference was found in the risk of BCSD (*p*=0.291) and OCSD (*p*=0.084) between the two groups (Figure 3). These results suggested that the number of prior cancers did not affect the prognosis of patients with P-TNBC.

### 3.3. Prognostic Factors for P-TNBC Patients Based on Univariate Analysis by CIF

Next, we further explored the prognostic factors of BCSD and OCSD in patients with P-TNBC via the CIF method. The results of the cumulative incidences of BCSD and OCSD at 1 year, 3 years, and 5 years are presented in Table 3. Among patients with P-TNBC, a total of 682 (59.3%) patients died from breast cancer (BCSD) and 469 (40.7%) patients died from other causes (OCSD) (Table 3). The result showed that the cumulative incidences of BCSD at 1 year, 3 years, and 5 years in patients with P-TNBC were 21.7%, 80.4%, and 96.8%, respectively. And the 1-year, 3-year, and 5-year cumulative incidences of OCSD in patients with P-TNBC were 22.6%, 66.5%, and 93.6%, respectively. Furthermore, we found that P-TNBC patients younger than 40 years or older than 80 years; black race; single status; advanced differentiation grade; advanced T stage; advanced N stage; receiving mastectomy, chemotherapy, or no radiotherapy were accompanied by high cumulative incidences of BCSD. Additionally, we found that patients with P-TNBC; older than 80 years; with single status; with T3 stage; and not receiving mastectomy treatment or chemotherapy or radiotherapy were accompanied by high cumulative incidences of OCSD. The site of laterality had no statistical significance in the risk of BCSD and OCSD.

### 3.4. Independent Prognostic Factors for P-TNBC Patients by the Fine and Gray Model

After the univariate analysis of CIF, the proportional distribution risk model of the Fine and Gray method was used to conduct multivariate analysis of BCSD and OCSD in patients with P-TNBC (Table 4). Age in diagnosis, tumor differentiation grade, T stage, and N stage were proven to be independent predictive factors of BCSD. The results of our study showed that, for patients with P-TNBC, subjects of 20–39 years had more probable BCSD (50–59 vs. 20–39: SHR = 0.67, *p*=0.041; 60–69 vs. 20–39: SHR = 0.65, *p*=0.028) in comparison with those with other age bands. Moreover, when compared with patients with grade I tumors, those with advanced differentiation grades had worse BCSD (III vs. I : SHR = 3.58, *p*=0.040; IV vs. I : SHR = 4.42, *p*=0.020). In addition, patients with advanced T stage tended to have a higher risk of BCSD when compared to those with T1 stage (T2 vs. T1 : SHR = 2.13, *p* < 0.001; T3 vs. T1 : SHR = 3.80, *p* < 0.001; T4 vs. T1 : SHR = 4.67, *p* < 0.001) tumor and subjects with advanced *N* status were likely to have better BCSD in comparison with those with N0 stage (N1 vs. N0 : SHR = 1.84, *p* < 0.001; *N*2 vs. N0 : SHR = 2.75, *p* < 0.001; N3 vs. N0 : SHR = 4.22, *p* < 0.001) tumor (Table 4).

When it came to OCSD, age in diagnosis, marital status, T stage, radiotherapy status, chemotherapy status, and surgery methods were proven to be independent predictive factors of OCSD (Table 4). Married patients with P-TNBC and those who underwent BCS tended to have a higher risk of OCSD (single vs. married: SHR = 0.49, *p*=0.014; mastectomy vs. BCS : SHR = 0.63, *p* < 0.001). Patients of 20–39 years had a lower risk of OCSD (70–79 vs. 20–39: SHR = 3.84, *p*=0.008; 80+ vs. 20–39: SHR = 5.79, *p*=0.001). In addition, patients with advanced *T* stage tended to have a higher risk of OCSD (T2 vs. T1 : SHR = 1.46, *p* < 0.001; T3 vs. T1 : SHR = 1.95, *p*=0.001; T4 vs. T1 : SHR = 2.12, *p*=0.002). Patients with radiotherapy or chemotherapy were more likely to have worse OCSD than those without radiotherapy or chemotherapy (yes vs. no : SHR 1.56, *p* < 0.001; yes vs. no : SHR 2.12, *p* < 0.001) (Table 4).

### 3.5. Comparison of the Prognosis for Patients with P-TNBC in Different Treatment Modes

**Figure 4:**
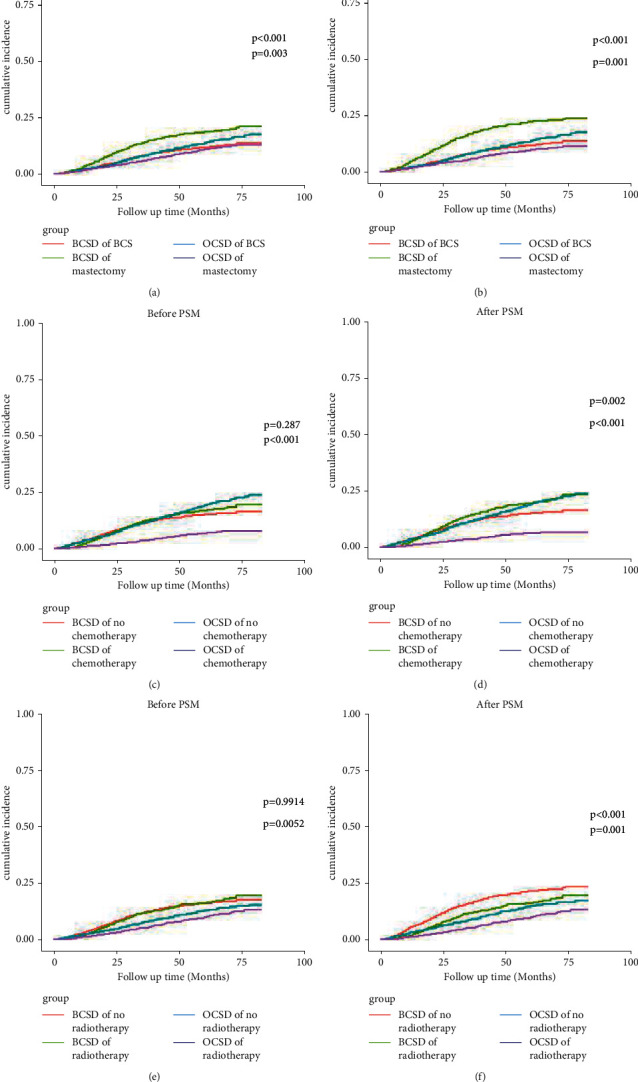
Cumulative incidence function analysis of P-TNBC patients with different treatments before and after PSM analysis. P-TNBC: triple-negative breast cancer with prior cancers; BCSD: breast cancer-specific death; OCSD: other cause-specific death; PSM: propensity score matching. *p* value <0.05 was considered statistically significant.

Through univariate CIF analysis, we found that chemotherapy and radiotherapy were not prognostic factors in patients with P-TNBC, whereas after PSM, the result came as the opposite.  Figure 4 shows the visualization of the results of univariate CIF analysis before and after PSM. As shown in Figure 4, whether before and after PSM, patients who received mastectomy were considered more likely to develop BCSD (*p* < 0.001, *p* < 0.001) (Figures 4(a) and 4(b)). Additionally, before PSM, the results showed that there was no difference in the impact of chemotherapy or radiotherapy on the risk of BCSD (*p*=0.287, *p*=0.991) (Figures 4(b) and 4(e)). However, after PSM, the results showed that subjects who received chemotherapy had worse BCSD, whereas those who received radiotherapy had improved prognosis (*p*=0.002, *p* < 0.001) (Figures 4(d) and 4(f)). From the perspective of OCSD risk, patients who had received BCS, no radiotherapy, or chemotherapy had more probable OCSD risk regardless of PSM analysis (Figures 4(a)–4(f)). To sum up, the above results indicated that the prognosis of patients with P-TNBC could be affected by surgical methods, chemotherapy, and radiotherapy status.

## 4. Discussion

The focus of this study was to investigate the prognostic factors of BCSD and OCSD risk in patients of SEER registries diagnosed with P-TNBC from 2010 to 2016 and the influence of different treatment modes on their prognosis. To our knowledge, this was the first and largest population-based study to explore this question.

Firstly, we found that prior cancer was a poor prognostic factor for BCSD and OCSD in patients with TNBC and the number of prior cancers did not affect the prognosis of patients with P-TNBC. Next, the cumulative incidences of BCSD and OCSD at 1 year, 3 years, and 5 years were 21.7%, 80.4%, 96.8%, respectively, and 22.6%, 66.5%, and 93.6%, respectively. Then, after univariate and multivariate competitive risk analysis, the results showed that younger age, advanced differentiation grade, advanced T stage, and advanced N stage had the higher risk of BCSD in patients with P-TNBC. However, surgery methods and radiotherapy or chemotherapy status were not related to the incidence of BCSD. Finally, to further confirm the effect of surgical methods, radiotherapy, and chemotherapy on BCSD for patients with P-TNBC, we combined PSM analysis with CIF competitive risk analysis and found that patients with P-TNBC who received BCS, radiotherapy, or chemotherapy had lower incidences of BCSD. To sum up, surgery methods, radiotherapy, and chemotherapy should be taken into account when assessing the risk in BCSD for patients with P-TNBC regardless of age in diagnosis, tumor differentiation grade, T stage, and N stage, which could help clinicians make more accurate treatment plans for P-TNBC patients.

Previous studies had shown that age, race, and prior cancer types could influence the incidence of second primary breast cancer (SPBC) [17]. For example, second primary cancer (SPBC) was more common in older (50 years old) women or White race patients with initial cutaneous melanoma than that in the general female population. In addition, Asian-pacific Islander (API) women with cancers of the uterus, ovary, bladder, or kidney were more likely to develop SPBC than the general population [17]. Patients with prior cancer had unfavorable overall survival [7], and prior cancer was proved to provide an inferior overall survival but a superior breast cancer-specific survival for patients with advanced breast cancer [18]. In the current study, similar conclusions were reached for patients with P-TNBC. We found that prior cancer was a poor prognostic factor for patients with P-TNBC, who were more likely to have worse BCSD and OCSD than those with NP-TNBC. Additionally, after divided patients with P-TNBC into one prior cancer cohort and two or more prior cancers cohort according to the number of prior cancer, we found that the number of cancer histories did not affect the probability of BCSD and OCSD for patients with P-TNBC.

Currently, with recent advances in research on early detection and treatment of breast cancer, the incidence of BCSD had decreased dramatically in the developed countries [19, 20]. Nevertheless, a corollary of reduced mortality was a greater likelihood of other benign or malignant diseases, such as secondary primary cancers and cardiovascular diseases [21]. Competitive risk events were common in clinical studies, especially in cancer-related research. However, traditional survival calculations, such as the Kaplan–Meier method and Cox regression model, would result in the estimation bias resulting from OCSD and increase the rough incidence of related events and overestimate their corresponding risk [22, 23]. In 1988, Gray proposed the CIF test to compare competitive risks [14]. In 1999, Fine and Gray proposed the comparative example distributed risk model for competitive projects [15].

Next, the univariate CIF and multivariate Fine and Gray competitive risk analysis method were utilized to select the independent prognostic factors of BCSD and OCSD in patients with P-TNBC. Patients of 20–39 years were found to have a higher risk of BCSD, whereas the risk of OCSD was lower. Studies had shown that age was associated with an increase in immune dysfunction and affected the prognosis of patients with TNBC [24]. Aapro and Wildiers also proved that elder patients with TNBC had better prognosis than younger patients [25]. In addition, it had been documented that the prognosis of Black/African American patients with TNBC was worse than that of White/European patients from the perspectives of tumor gene localization, immune microenvironment, and tumor lymphocyte infiltration [26, 27]. Relevant studies had reported that the risk of marital status-related death depended on race/ethnicity, and only White and Asian/Pacific Islanders women were found to have a marital advantage in long-term survival for patients with TNBC [28]. However, in the current study, we found that race, as well as marital status, were not independent prognostic factors for BCSD in patients with P-TNBC. The most plausible explanation was that the target population of previous research was women with primary TNBC, while in our study the target population was participants with P-TNBC, the history of which was proven to be an adverse prognostic factor for patients with P-TNBC in our study. Then, prior cancer weakened the prognostic impact originating from racial differences. Moreover, in the current study, we found that P-TNBC patients who underwent mastectomy tended to have worse prognosis than those who underwent BCS. This was consistent with the result of a previous study which had proven the 5-year overall survival of BCS was better than that of mastectomy (92.9% vs. 89.7%) [29].

Radiotherapy significantly improved the long-term prognosis of TNBC patients after BCS [30–32]. In our current study, radiotherapy was not associated with BCSD for P-TNBC patients who underwent BCS. The underlying reason might be that the follow-up visits for patients with prior cancer were always frequently, regularly, and more likely to be firstly diagnosed with early breast cancer. Then, the administration of radiotherapy did not affect the prognosis for patients with prior cancer. Currently, chemotherapy, polyadenosine diphosphate, troP-2 targeted antibody-drug conjugates, and immunotherapy were proven to be effective systemic treatments for patients with TNBC [33–36]. However, it remains indistinct in the effect of polyadenosine diphosphate, troP-2 targeted antibody-drug conjugates, and immunotherapy for patients with P-TNBC.

In addition, this study has limitations. Firstly, there are still some patients without completed demographic and clinical information, which may result in selection bias, in the SEER database. Secondly, the survival outcomes may be impacted by the status of cancer histories and fertility. However, due to the lack of relevant authority, a detailed type of prior cancer and data of fertility status could not be obtained in our study. Thirdly, since the “No” subgroup in the chemotherapy field contains a part of “unknown” patients who cannot be identified and excluded from this study, it may affect our results; the same is true in the radiation field. Fourth, although the specific protocol of the radiation therapy field and the order relationship between radiation therapy and surgery were recorded in the SEER database, there was a large difference in the number of patients among the subgroups, which could not be further analyzed. Last but not least, as a retrospective cohort population, inevitable selection bias may influence the conclusions. In the future, larger prospective randomized controlled trials are necessary to identify risk factors.

## 5. Conclusions

For patients with TNBC, prior cancer was found to be an adverse prognostic factor for BCSD and OCSD, while the number of prior cancers was not associated with the prognosis for patients with P-TNBC. In conclusion, our study demonstrated that prior cancer was related to the worse BCSD and OCSD rate and could be identified as a reliable survival predictor for patients with nonmetastatic TNBC. This study could provide some reference value for the treatment models of TNBC patients with prior cancer in the future. Randomized controlled clinical trials with long follow-up times are still needed to provide a high level of evidence on the disadvantages of prior cancer for patients with TNBC.

## Figures and Tables

**Table 1:** Baseline characteristics of patients diagnosed with TNBC before and after propensity score matching.

Characteristics	Before PSM			After PSM		
	No prior cancer	Prior cancer	*p* value	No prior cancer	Prior cancer	*p* value
Total	*n* = 24219 (%)	*n* = 5375 (%)		*n* = 5375 (%)	*n* = 5375 (%)	
Age			<0.001			0.985
20–39	2265 (9.4)	174 (3.2)		161 (3.0)	174 (3.2)	
40–49	4822 (19.9)	582 (10.8)		592 (11.0)	582 (10.8)	
50–59	6456 (26.7)	1220 (22.7)		1210 (22.5)	1220 (22.7)	
60–69	5791 (23.9)	1541 (28.7)		1551 (28.9)	1541 (28.7)	
70–79	3225 (13.3)	1185 (22.0)		1186 (22.1)	1185 (22.0)	
80+	1660 (6.9)	673 (12.5)		675 (12.6)	673 (12.5)	

Race			<0.001			0.923
Black	4877 (20.1)	990 (18.4)		982 (18.3)	990 (18.4)	
White	1920 (7.9)	340 (6.3)		332 (6.2)	340 (6.3)	
Other	17422 (71.9)	4045 (75.3)		4061 (75.6)	4045 (75.3)	

Marital			<0.001			0.629
Married	13987 (57.8)	2850 (53.0)		2824 (52.5)	2850 (53.0)	
Single	10232 (42.2)	2525 (47.0)		2551 (47.5)	2525 (47.0)	

Laterality			0.436			0.44
Left	12440 (51.4)	2793 (52.0)		2752 (51.2)	2793 (52.0)	
Right	11779 (48.6)	2582 (48.0)		2623 (48.8)	2582 (48.0)	

Grade			<0.001			0.247
I	422 (1.7)	162 (3.0)		129 (2.4)	162 (3.0)	
II	3994 (16.5)	1164 (21.7)		1157 (21.5)	1164 (21.7)	
III	19638 (81.1)	4016 (74.7)		4059 (75.5)	4016 (74.7)	
IV	165 (0.7)	33 (0.6)		30 (0.6)	33 (0.6)	

T status			<0.001			0.706
T1	10852 (44.8)	3133 (58.3)		3128 (58.2)	3133 (58.3)	
T2	10418 (43.0)	1800 (33.5)		1835 (34.1)	1800 (33.5)	
T3	1947 (8.0)	272 (5.1)		254 (4.7)	272 (5.1)	
T4	1002 (4.1)	170 (3.2)		158 (2.9)	170 (3.2)	

N status			<0.001			0.441
N0	16141 (66.6)	4100 (76.3)		4109 (76.4)	4100 (76.3)	
N1	5572 (23.0)	863 (16.1)		891 (16.6)	863 (16.1)	
N2	1460 (6.0)	227 (4.2)		216 (4.0)	227 (4.2)	
N3	1046 (4.3)	185 (3.4)		159 (3.0)	185 (3.4)	

Surgery			<0.001			0.904
BCS	12735 (52.6)	1981 (36.9)		1974 (36.7)	1981 (36.9)	
Mastectomy	11484 (47.4)	3394 (63.1)		3401 (63.3)	3394 (63.1)	

Radiotherapy			<0.001			0.526
No	11353 (46.9)	3605 (67.1)		3573 (66.5)	3605 (67.1)	
Yes	12866 (53.1)	1770 (32.9)		1802 (33.5)	1770 (32.9)	

Chemotherapy			<0.001			0.797
No	5615 (23.2)	2120 (39.4)		2106 (39.2)	2120 (39.4)	
Yes	18604 (76.8)	3255 (60.6)		3269 (60.8)	3255 (60.6)	

*Note.* TNBC: triple-negative breast cancer with prior cancer; BCS: breast-conserving therapy. *p* value <0.05 was considered statistically significant.

**Table 2:** Baseline characteristics of patients diagnosed with P-TNBC before and after propensity score matching.

Characteristics	Before PSM			After PSM		
	One prior cancer	Two or more prior cancers	*p* value	One prior cancer	Two or more prior cancers	*p* value
Total	*n* = 4584 (85.3%)	*n* = 791 (14.7%)		*n* = 791 (50%)	*n* = 791 (50%)	
Age						
20–39	164 (3.6)	10 (1.3)	<0.001	9 (1.1)	10 (1.3)	0.949
40–49	533 (11.6)	49 (6.2)		56 (7.1)	49 (6.2)	
50–59	1055 (23.0)	165 (20.9)		171 (21.6)	165 (20.9)	
60–69	1327 (28.9)	214 (27.1)		215 (27.2)	214 (27.1)	
70–79	957 (20.9)	228 (28.8)		213 (26.9)	228 (28.8)	
80+	548 (12.0)	125 (15.8)		127 (16.1)	125 (15.8)	

Race						
Black	857 (18.7)	133 (16.8)	0.001	125 (15.8)	133 (16.8)	0.647
White	312 (6.8)	28 (3.5)		23 (2.9)	28 (3.5)	
Other`	3415 (74.5)	630 (79.6)		643 (81.3)	630 (79.6)	

Marital						
Married	2447 (53.4)	403 (50.9)	0.22	394 (49.8)	403 (50.9)	0.687
Single	2137 (46.6)	388 (49.1)		397 (50.2)	388 (49.1)	

Laterality						
Left	2403 (52.4)	390 (49.3)	0.114	393 (49.7)	390 (49.3)	0.92
Right	2181 (47.6)	401 (50.7)		398 (50.3)	401 (50.7)	

Grade						
I	140 (3.1)	22 (2.8)	0.805	16 (2.0)	22 (2.8)	0.808
II	985 (21.5)	179 (22.6)		180 (22.8)	179 (22.6)	
III	3432 (74.9)	584 (73.8)		589 (74.5)	584 (73.8)	
IV	27 (0.6)	6 (0.8)		6 (0.8)	6 (0.8)	

T status						
T1	2643 (57.7)	490 (61.9)	0.135	516 (65.2)	490 (61.9)	0.162
T2	1556 (33.9)	244 (30.8)		238 (30.1)	244 (30.8)	
T3	239 (5.2)	33 (4.2)		20 (2.5)	33 (4.2)	
T4	146 (3.2)	24 (3.0)		17 (2.1)	24 (3.0)	

N status						
N0	3435 (74.9)	665 (84.1)	<0.001	676 (85.5)	665 (84.1)	0.452
N1	781 (17.0)	82 (10.4)		83 (10.5)	82 (10.4)	
N2	205 (4.5)	22 (2.8)		19 (2.4)	22 (2.8)	
N3	163 (3.6)	22 (2.8)		13 (1.6)	22 (2.8)	

Surgery						
BCS	1734 (37.8)	247 (31.2)	<0.001	255 (32.2)	247 (31.2)	0.705
Mastectomy	2850 (62.2)	544 (68.8)		536 (67.8)	544 (68.8)	

Radiotherapy						
No	3016 (65.8)	589 (74.5)	<0.001	597 (75.5)	589 (74.5)	0.685
Yes	1568 (34.2)	202 (25.5)		194 (24.5)	202 (25.5)	

Chemotherapy						
No	1719 (37.5)	401 (50.7)	<0.001	393 (49.7)	401 (50.7)	0.725
Yes	2865 (62.5)	390 (49.3)		398 (50.3)	390 (49.3)	

*Note*. P-TNBC: triple-negative breast cancer with prior cancer; BCS: breast-conserving therapy; PSM: propensity score matching.

**Table 3:** Cumulative incidence function analysis of death causes in woman patients with P-TNBC.

Characteristics	BCSD					OCSD				
	Event	1 year (%)	3 years (%)	5 years (%)	*p* value	Event	1 year (%)	3 years (%)	5 years (%)	*p* value
Total	*n* = 682(59.3)	21.7	80.4	96.8	<0.001	*n* = 469(40.7)	22.6	66.5	93.6	<0.001
Age					<0.001					<0.001
20–39	32 (4.7)	4.0	17.2	22.6		4 (0.9)	0.6	2.1	3.1	
40–49	82 (12.0)	2.9	12.2	16.9		26 (5.5)	0.7	3.0	5.8	
50–59	140 (20.5)	1.7	11.3	14.8		57 (12.2)	1.2	3.6	6.1	
60–69	159 (23.3)	1.9	9.9	13.0		92 (19.6)	1.8	5.1	8.2	
70–79	155 (22.7)	3.1	12.1	17.1		138 (29.4)	2.1	9.1	16.0	
80+	114 (16.7)	5.8	15.7	20.9		152 (32.4)	5.2	17.1	30.4	

Race					0.023					0.113
Black	149 (21.8)	3.2	15.4	18.1		95 (20.3)	2.5	7.6	13.8	
White	497 (72.9)	2.8	11.1	15.6		354 (75.5)	1.9	6.7	11.5	
Other	36 (5.3)	0.9	10.6	14.8		20 (4.3)	0.9	6.5	8.7	

Marital					0.001					<0.001
Married	321 (47.1)	2.0	10.4	14.5		186 (39.7)	1.5	5.4	8.6	
Single	361 (52.9)	3.6	13.6	17.8		283 (60.3)	2.5	8.4	15.3	

Laterality					0.629					0.430
Left	350 (51.3)	2.0	11.9	15.6		254 (54.2)	1.8	6.6	12.0	
Right	332 (48.7)	2.9	11.9	16.6		215 (45.8)	2.2	7.0	11.5	

Grade					<0.001					0.127
I	7 (1.0)	0.6	4.2	4.2		12 (2.6)	1.2	5.1	9.3	
II	111 (16.3)	1.9	8.1	12.3		87 (18.6)	1.8	5.6	10.3	
III	557 (81.7)	3.1	13.2	17.6		364 (77.6)	2.1	7.2	12.2	
IV	7 (1.0)	3.0	19.4	23.3		6 (1.3)	3.0	13.1	23.0	

T status					<0.001					0.002
T1	211 (30.9)	1.2	6.0	8.4		241 (51.4)	1.5	5.7	10.5	
T2	300 (44.0)	3.3	15.8	21.6		174 (37.1)	2.3	7.8	13.3	
T3	94 (13.8)	12.3	33.1	43.2		33 (7.0)	3.3	9.7	14.7	
T4	77 (11.3)	11.8	46.1	55.2		21 (4.5)	5.3	12.4	13.4	

N status					<0.001					0.716
N0	341 (50.0)	1.6	7.9	10.5		352 (75.1)	2.0	6.9	11.5	
N1	167 (24.5)	4.1	17.3	25.1		73 (15.6)	1.2	5.9	12.7	
N2	79 (11.6)	10.6	33.8	41.9		25 (5.3)	3.5	8.3	13.2	
N3	95 (13.9)	13.6	46.9	59.4		19 (4.1)	3.8	8.5	10.5	

Surgery					<0.001					0.003
BCS	179 (26.2)	1.6	8.1	11.8		201 (42.9)	2.2	8.2	13.5	
Mastectomy	503 (73.8)	3.5	14.1	18.5		268 (57.1)	1.9	6.0	10.8	

Radiotherapy					0.991					0.005
No	456 (66.9)	3.3	12.0	16.1		341 (72.7)	2.4	7.6	12.8	
Yes	226 (33.1)	1.7	11.8	16.0		128 (27.3)	1.2	5.2	9.6	

Chemotherapy					0.287					<0.001
No	263 (38.6)	3.7	11.4	15.0		315 (67.2)	3.6	11.6	19.0	
Yes	419 (61.4)	2.2	12.3	16.8		154 (32.8)	0.9	3.6	6.7	

*Note*. P-TNBC: triple-negative breast cancer with prior cancer; BCS: breast-conserving therapy; BCSD: breast cancer-specific death; OCSD: other cause-specific death. *p* value <0.05 was considered statistically significant.

**Table 4:** The Fine and Gray proportional subdistribution hazard model for BCSD and OCSD in woman patients with P-TNBC.

Characteristics	BCSD			OCSD		
	SHR	95% CI	*p* value	SHR	95% CI	*p* value
Age						
20–39	1			1		
40–49	0.73	0.49–1.11	0.140	1.76	0.61–1.76	0.290
50–59	0.67	0.46–0.98	0.041	1.89	0.68–1.89	0.220
60–69	0.65	0.44–0.95	0.028	2.38	0.87–2.38	0.092
70–79	0.84	0.57–1.23	0.360	3.84	1.41–3.84	0.008
80+	0.91	0.60–1.37	0.630	5.79	2.13–5.79	0.001

Race						
Black	1			—		
White	0.87	0.72–1.05	0.140	—	—	—
Other	0.75	0.53–1.07	0.120	—	—	—

Marital						
Married	1			1		
Single	0.92	0.79–1.08	0.330	0.79	0.65–0.79	0.014

Grade						
I	1			—		
II	2.06	0.90–4.21	0.089	—	—	—
III	3.58	1.04–4.70	0.040	—	—	—
IV	4.42	1.22–10.56	0.020	—	—	—

T status						
T1	1			1		
T2	2.13	1.71–2.48	<0.001	1.46	1.19–1.46	<0.001
T3	3.80	2.70–4.76	<0.001	1.95	1.33–1.95	0.001
T4	4.67	3.21–6.10	<0.001	2.12	1.31–2.12	0.002

N status						
N0	1			—		
N1	1.84	1.51–2.24	<0.001	—	—	—
N2	2.75	2.09–3.63	<0.001	—	—	—
N3	4.22	3.24–5.49	<0.001	—	—	—

Surgery						
BCS	1			1		
Mastectomy	1.17	0.98–1.40	0.480	0.63	0.51–0.63	<0.001

Radiotherapy						
No	—			1		
Yes	—	—	—	1.56	1.22–1.56	<0.001

Chemotherapy						
No	—			1		
Yes	—	—	—	2.12	1.71–2.12	<0.001

*Note*. P-TNBC: triple-negative breast cancer with prior cancers; BCS: breast-conserving therapy; BCSD: breast cancer-specific death; OCSD: other cause-specific death; SHR: subdistribution hazard ratio; 95%CI: 95% confidence interval. *p* value <0.05 was considered statistically significant.

## Data Availability

Data of this study are derived from the Surveillance, Epidemiology, and End Results Program tumor registries for the creation and maintenance of the open database.
